# Measuring the complexity of directed graphs: A polynomial-based approach

**DOI:** 10.1371/journal.pone.0223745

**Published:** 2019-11-14

**Authors:** Matthias Dehmer, Zengqiang Chen, Frank Emmert-Streib, Shailesh Tripathi, Abbe Mowshowitz, Alexei Levitchi, Lihua Feng, Yongtang Shi, Jin Tao

**Affiliations:** 1 Institute for Intelligent Production, Faculty for Management, University of Applied Sciences Upper Austria, Steyr, Austria; 2 College of Artificial Intelligence, Nankai University, Tianjin, China; 3 Department of Biomedical Computer Science and Mechatronics, UMIT - The Health and Lifesciences University, A-6060 Hall in Tyrol, Austria; 4 Predictive Medicine and Data Analytics Lab, Department of Signal Processing, Tampere University, Tampere, Finland; 5 Institute of Biosciences and Medical Technology, Tampere, Finland; 6 Department of Computer Science, The City College of New York (CUNY), 138th Street at Convent Avenue, New York, United States of America; 7 School of Mathematics and Statistics, Central South University, Changsha, China; 8 Center for Combinatorics and LPMC, Nankai University, Tianjin, China; 9 Department of Electrical Engineering and Automation, Aalto University, Espoo, Finland; 10 College of Engineering, Peking University, Beijing, China; Universidad Rey Juan Carlos, SPAIN

## Abstract

In this paper, we define novel graph measures for directed networks. The measures are based on graph polynomials utilizing the out- and in-degrees of directed graphs. Based on these polynomial, we define another polynomial and use their positive zeros as graph measures. The measures have meaningful properties that we investigate based on analytical and numerical results. As the computational complexity to compute the measures is polynomial, our approach is efficient and can be applied to large networks. We emphasize that our approach clearly complements the literature in this field as, to the best of our knowledge, existing complexity measures for directed graphs have never been applied on a large scale.

## 1 Introduction

Graph complexity measures have been studied extensively [1–4]. Although a large number of complexity measures have been defined, few deal specifically with directed graphs. However, many real-world networks such as transportation networks [5] and biological networks [6] are directed graphs whose edges express critical interactions, flows and so forth. Examples of complexity measures for undirected graphs include treewidth [2], cycle rank [2] and numerous so-called topological indices, see [4, 7]. Some of the classical graph complexity indices like the distance-based Wiener index [8] or the graph entropy measure based on vertex orbits due to Mowshowitz [9] can be computed for directed graphs as well. For example, Knor et al. [10] studied the Wiener Index on directed graphs. Other classical and distance-based measures like the Szeged index [11] could also be applied to directed graphs. But to the best of our knowledge, there is no body of literature that focuses on comparing structural graph measures for undirected and directed graphs.

Measures for analyzing directed graphs [12] include DAG-width [3], directed treewidth [13] and girth [1]. Treewidth and directed treewidth are both based on a game-theory applied to special graph decompositions. It might be difficult to apply these measures to large real-world networks. Also, the girth of a directed graph has been defined as the minimum length of a directed cycle [1]. If the graph is acyclic, the girth is infinite [1]. Another technique is due to Bertz et al. [14]; they investigate the complexity of digraphs by identifying all possible subgraphs with a certain number of vertices representing patterns such as trees, paths, rings etc. Degree sequences are then used to quantify the complexity or diversity of the digraphs [14]. Hunter and Kreutzer [15] investigate the meaning of several methods for determining the complexity of directed graphs and point out differences between undirected and directed graphs. Berwanger and Grädel [16] use special tree-decompositions and define the graph measure entanglement and its relationship to treewidth. Estrada and Hatano [17] define the measures reciprocity and returnability based on eigenvalues of special graph-theoretical matrices. Heterogeneity measures, interpreted as irregularity based on differences between in-degrees and out-degrees, have been developed by Ye et al. [18]. We emphasize that in this paper, we put the emphasis on examining complexity measures for analyzing complex networks. Another important branch of Quantitative Graph Theory [19] relates to measure the similarity between networks. See [20] for an up-to-date review to survey this area.

In this paper, we propose an approach that departs from the contributions sketched above. Based on the occurrences of out- and in-degrees of directed graphs, we define certain graph polynomials. We show that every directed graph can be characterized by an out- and in-degree polynomial. In order to obtain positive zeros, we define modified graph polynomials and show they must possess a unique, positive zero in the interval (0, 1), depending on certain parameters. So, we analyze properties of these polynomials and prove interrelations between their zeros. Based on these zeros, we define graph complexity measures and investigate issues such as the correlation between the measures and the homogeneity of the zeros which are associated with a graph.

## 2 Methods

### 2.1 New complexity measures for directed graphs

In this section, we introduce some preliminaries. The directed graphs [21] considered here are without loops and multiple edges.

**Definition 2.1** *Let* *G* = (*V*, *E*), *E* ⊆ *V* × *V*, |*V*| < ∞ *be a directed graph*.

$$N^{+}\left(v\right)\;≔\{\tilde{v}\in V\backslash \left\{v\right\}|\left(v,\;\tilde{v}\right)\in E\}\;is\;the\;set\;of\;out-neighbors\;of\;v,$$ (1)

$$N^{-}\left(v\right)\;≔\{\tilde{u}\in V\backslash \left\{v\right\}|\left(\tilde{u},v\right)\in E\}\;is\;the\;set\;of\;in-neighbors\;of\;v,$$ (2)

$$\delta_{out}\left(v\right)\;≔|N^{+}\left(v\right)|,$$

$$\delta_{in}\left(v\right)\;≔|N^{-}\left(v\right)|.$$ (3)

**Definition 2.2** *Let* *G* = (*V*, *E*), *E* ⊆ *V* × *V*, |*V*| < ∞ *be a directed graph*.

$$\begin{array}{lll} N_{0}^{+} & ≔\{v\in V|\delta_{out}\left(v\right)=0\}, & \left(4\right)\\ N_{0}^{+} & ≔\{v\in V|\delta_{out}\left(v\right)=1\}, & \left(5\right)\\ \vdots & \;\vdots & \left(6\right)\\ N_{\delta_{out}^{max}}^{+} & ≔\{v\in V|\delta_{out}\left(v\right)=\delta_{out}^{max}\}. & \left(7\right) \end{array}$$

**Definition 2.3** *Let* *G* = (*V*, *E*), *E* ⊆ *V* × *V*, |*V*| < ∞ *be a directed graph*.

$$\begin{array}{lll} N_{0}^{-} & ≔\{v\in V|\delta_{in}\left(v\right)=0\}, & \left(8\right)\\ N_{1}^{-} & ≔\{v\in V|\delta_{in}\left(v\right)=1\}, & \left(9\right)\\ \vdots & \;\vdots & \left(10\right)\\ N_{\delta_{in}^{max}}^{-} & ≔\{v\in V|\delta_{in}\left(v\right)=\delta_{in}^{max}\}. & \left(11\right) \end{array}$$

Now, we define two special graph polynomials with real coefficients. Note that the coefficients capture structural information of the given graph.

**Definition 2.4** *We define the coefficients of the graph polynomial* *P*_*G*,*out*_(*x*) *by*

$$\begin{array}{lll} a_{k}^{out} & ≔\left|N_{\delta_{out}^{max}}^{+}\right|, & \left(12\right)\\ a_{k-1}^{out} & ≔\left|N_{\delta_{out}^{max}-1}^{+}\right|, & \left(13\right)\\ \vdots & \;\vdots & \left(14\right)\\ a_{1}^{out} & ≔\left|N_{1}^{+}\right|, & \left(15\right)\\ a_{0}^{out} & ≔\left|N_{0}^{+}\right|. & \left(16\right) \end{array}$$

*Finally*, $P_{G,out}\left(x\right)\;≔\;a_{k}^{out}x^{\delta_{out}^{max}}+\cdots +\;a_{1}^{out}x\;+\;a_{0}^{out}x^{0}$.

Similarly,

**Definition 2.5** *we define the coefficients of the graph polynomial* *P*_*G*,in_(*x*) *by*

$$\begin{array}{lll} a_{k}^{in} & ≔\left|N_{\delta_{in}^{max}}^{+}\right|, & \left(17\right)\\ a_{k-1}^{in} & ≔\left|N_{\delta_{in}^{max}-1}^{+}\right|, & \left(18\right)\\ \vdots & \;\vdots & \left(19\right)\\ a_{1}^{in} & ≔\left|N_{1}^{+}\right|, & \left(20\right)\\ a_{0}^{in} & ≔\left|N_{0}^{+}\right|. & \left(21\right) \end{array}$$

*Finally*, $P_{G,in}\left(x\right)\;≔\;a_{k}^{in}x^{\delta_{in}^{max}}\;+\cdots +\;a_{1}^{in}x\;+\;a_{0}^{in}x^{0}$.

Obviously *P*_*G*,out_(*x*) and *P*_*G*,in_(*x*) have no positive zeros since their sequences of coefficients have no sign changes. This follows from Descartes’ Rule of Signs, see [22]. In the following, we establish the conditions under which two associated polynomials have a unique, positive zero ∈ (0, 1).

**Theorem 2.1** *Let* *G* = (*V*, *E*) *be a directed graph. Now we define the polynomials*

$$P^{*}{}_{G,out}\left(x\right)\;≔\;\alpha_{out}-P_{G,out}\left(x\right),$$ (22)

$$P^{*}{}_{G,in}\left(x\right)\;≔\;\alpha_{in}-P_{G,in}\left(x\right).$$ (23)

*There exist parameters* $\alpha_{out}\in \mathbb{R}$ *and* $\alpha_{in}\in \mathbb{R}$, *such that the polynomials* P*_G,*out*_(*x*) *and* P*_G,*in*_(*x*) *have a unique*, *positive zero* *δ*_*out*_(*G*) *and* *δ*_*in*_(*G*), *respectively*. *In fact*, *the values of* *δ*_*out*_(*G*) *and* *δ*_*in*_(*G*) *depend on* *α*_*out*_ *and* *α*_*in*_, *respectively*.

**Proof**: In order to simplify the notation, we write the polynomial as

$$\begin{array}{c} P_{G}\left(x\right)=a_{k}x^{k}+\cdots +a_{1}x+a_{0},\;a_{i}\in Z. \end{array}$$ (24)

Also, we need to define

$$\begin{array}{c} P_{G}^{⋆}\left(x\right)=\alpha -P_{G}\left(x\right)=\alpha -(a_{k}x^{k}+\cdots +a_{1}x+a_{0}). \end{array}$$ (25)

Observe that $\lim_{x\to \infty }P_{G}^{⋆}\left(x\right)=-\infty$. Assuming $P_{G}^{⋆}\left(0\right)>0$ yields the inequality

$$\begin{array}{c} \alpha -a_{0}>0\;\text{or}\;\alpha >a_{0}. \end{array}$$ (26)

Since the sequence of coefficients of $P_{G}^{⋆}\left(x\right)$ has only one sign change, Descartes’ Rule of Signs tells us that $P_{G}^{⋆}\left(x\right)$ has a unique zero *δ*. Now *δ* ∈ (0, 1) if

$$\begin{array}{c} P_{G}^{⋆}\left(1\right)=\alpha -(a_{k}+\cdots +a_{1}+a_{0})<0, \end{array}$$ (27)

and, finally

$$\begin{array}{c} \alpha <a_{k}+\cdots +a_{1}+a_{0}. \end{array}$$ (28)

Thus the inequalities (26) and (28) provide a range for *δ* ∈ (0, 1). It is evident that *δ* depends on the choice of *α* needed to satisfy the Inequalities (26) and (28). Finally, the theorem holds for the two different polynomials represented by the Eqs (22) and (23) along with the corresponding parameters *α*_out_ and *α*_in_.

In the following, we elaborate briefly on the problem of choosing *α*_out_ and *α*_in_. Again, consider the Inequalities (26) and (28), whose parameters can be real numbers or positive integers. If we choose real numbers, we get an infinite number of polynomials P*_G,out_(*x*) and P*_G,in_(*x*) whose roots lie in the interval (0, 1). If *α*_out_ and *α*_in_ are taken to be positive integers, the set of possible polynomials is finite. To determine the effect of the parameters on the roots, we appeal to the continuity theorem for complex and real polynomials, see [22]. This theorem states that the zeros of a polynomial are continuous functions of the coefficients of the polynomial, which mean a small change in the coefficients will cause only a small change in value of the zeros [22]. It seems to be unclear who was the first who proved the continuity theorem. Yet, it appears that a proof was already given by Weber in 1895, see [23]. Several other proofs of this statement have also been given independently, see [22].

Suppose, *G* is a directed graph and we wish to apply Theorem (2.1). To do this we have to determine the sets $\left\{\alpha_{\text{out}}^{\left[1\right]},\ldots ,\alpha_{\text{out}}^{\left[p\right]}\right\}$ and $\left\{\alpha_{\text{in}}^{\left[1\right]},\ldots ,\alpha_{\text{in}}^{\left[q\right]}\right\}$, if we choose positive integers. The following ordering can be obtained by permuting the indices

$$\begin{array}{c} \alpha_{\text{out}}^{\left[1\right]}<\alpha_{\text{out}}^{\left[2\right]}<\cdots \alpha_{\text{out}}^{\left[p\right]}, \end{array}$$ (29)

and

$$\begin{array}{c} \alpha_{\text{in}}^{\left[1\right]}<\alpha_{\text{in}}^{\left[2\right]}<\cdots \alpha_{\text{in}}^{\left[q\right]}. \end{array}$$ (30)

The sets of roots $\left\{\delta_{\text{out}}^{1}\left(G\right),\ldots ,\delta_{\text{out}}^{p}\left(G\right)\right\}$ and $\left\{\delta_{\text{in}}^{1}\left(G\right),\ldots ,\delta_{\text{in}}^{q}\left(G\right)\right\}$ in the interval (0, 1) can also be obtained. Applying the continuity theorem may lead to a simplification. For instance, we always choose the minimum value namely $\alpha_{\text{out}}^{\left[1\right]}\in Z$ and $\alpha_{\text{in}}^{\left[1\right]}\in Z$ satisfying the Inequalities (26) and (28). Consequently, we could reduce the problem to the zeros $\left\{\delta_{\text{out}}^{1}\left(G\right)\right\}$ and $\left\{\delta_{\text{in}}^{1}\left(G\right)\right\}$. In Section (3.5), we investigate numerically the effect of parameters *α*_out_ and *α*_in_ on the zeros of P*_G,out_(*x*) and P*_G,in_(*x*) numerically.

### 2.2 Graph complexity measures

In this section, we define some complexity measures on directed graphs based on the findings of Section (2.1). Recall the two polynomials, defined earlier, based on out- and in-degrees (see the Definitions (2.4), (2.5), and the modified polynomials with unique positive zeros in the interval (0, 1). Here, we argue that these zeros can serve as measures of the structural complexity of a directed graph. These measures are similar to those defined as a function of the eigenvalues of certain graph polynomials, see, e.g., [24, 25]. The eigenvalue based measures, represented by Eqs (33)–(36), have been defined with an eye to reducing their degeneracy, see also [24–26]. Degeneracy implies that a measure is unable to distinguish between non- isomorphic graphs, [4, 26], and is thus an undesirable property. Taking account of this we define the following measures.

**Definition 2.6**

$$I_{1}\left(G\right)=\delta_{out}\left(G\right),$$ (31)

$$I_{2}\left(G\right)=\delta_{in}\left(G\right),$$ (32)

$$I_{3}\left(G\right)=\frac{1}{2}\delta_{out}\left(G\right)+\frac{1}{2}\delta_{in}\left(G\right)$$ (33)

$$I_{4}\left(G\right)=\frac{1}{2}\sqrt{\delta_{out}\left(G\right)}+\frac{1}{2}\sqrt{\delta_{in}\left(G\right)},$$ (34)

$$I_{5}\left(G\right)=|\text{ln}\left(\delta_{out}\left(G\right)\right)\left|+\right|\text{ln}\left(\delta_{in}\right)\left(G\right)|,$$ (35)

$$I_{7}\left(G\right)=\frac{\left|E\right|}{{\left|V\right|}^{2}-\left|V\right|}$$ (36)

*δ*_out_(*G*) and *δ*_in_(*G*) are unique, positive zeros of the respective out- and in-degree polynomials. *I*_7_ is the well-known edge density [27].

### 2.3 Examples

**Fig 1:**
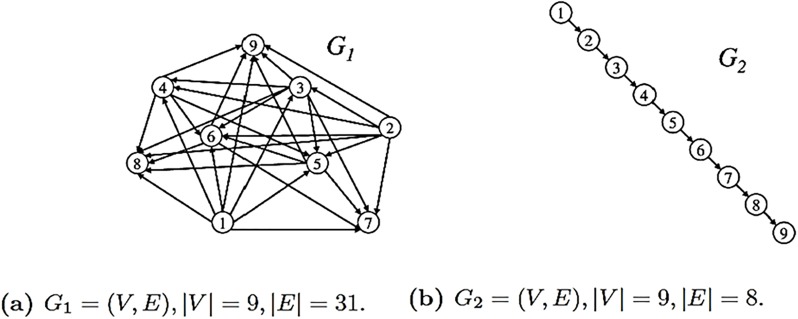
Two example graphs from $G_{1}$.

In the previous section, we briefly discussed how to find the parameters *α*_out_ and *α*_in_ by using Theorem (2.1). Now, we calculate the polynomials P*_G,out_(*x*) and P*_G,in_(*x*) as well as their roots in some special cases. Consider the graphs shown in Fig (1). For *G*_1_ we determine

$$\begin{array}{ll} N_{0}^{+} & =\{v_{7},v_{8},v_{9}\},\;N_{1}^{+}=\{\},\;N_{2}^{+}=\{\},\\ N_{3}^{+} & =\{v_{6}\},\;N_{4}^{+}=\{v_{4},v_{5}\},\;N_{5}^{+}=\{\},\\ N_{6}^{+} & =\{v_{3}\},\;N_{7}^{+}=\{v_{1},v_{2}\},\\ N_{0}^{-} & =\{v_{1},v_{2}\},\;N_{1}^{-}=\{\},\\ N_{2}^{-} & =\{v_{3}\},\;N_{3}^{-}=\{v_{4}\},\;N_{4}^{-}=\{v_{5}\},\\ N_{5}^{-} & =\{v_{6},v_{7}\},\;N_{6}^{-}=\{v_{8},v_{9}\}. \end{array}$$

and

$$\begin{array}{c} a_{7}^{\text{out}}=2,a_{6}^{\text{out}}=1,a_{5}^{\text{out}}=0,a_{4}^{\text{out}}=2,a_{3}^{\text{out}}=1,a_{2}^{\text{out}}=0,a_{1}^{\text{out}}=0,a_{0}^{\text{out}}=3, \end{array}$$

$$\begin{array}{c} a_{6}^{\text{in}}=2,a_{5}^{\text{in}}=2,a_{4}^{\text{in}}=1,a_{3}^{\text{in}}=1,a_{2}^{\text{in}}=1,a_{1}^{\text{in}}=0,a_{0}^{\text{in}}=2. \end{array}$$

From definitions (2.4), (2.5), we obtain

$$P_{G_{1},\text{out}}\left(x\right)=2x^{7}+x^{6}+2x^{4}+x^{3}+3,$$

$$P_{G_{1},\text{in}}\left(x\right)=2x^{6}+2x^{5}+x^{4}+x^{3}+x^{2}+2.$$

To determine the range of *α*_out_, we use the Inequalities (26) and (28) and infer

$$\begin{array}{c} \alpha_{\text{out}}>3\;\text{and}\;\alpha_{\text{out}}<2+2+1+1+1+2=9. \end{array}$$ (37)

According to Theorem (2.1), P*_G,out_(*x*) and P*_G,in_(*x*) have a unique positive zero in the interval (0, 1) if 3 < *α*_out_ < 9. If we choose positive integers, we obtain the set {4,5,6,7,8} as valid candidates. In Section (2.1) we explained that due to the continuity of the zeros, it makes sense to choose the minimum value of this set in order to calculate the zero. Thus,

$$\begin{array}{c} {P^{*}}_{G,\text{out}}\left(x\right)=\alpha_{\text{out}}-P_{G,out}\left(x\right)=4-\left(2x^{7}+x^{6}+2x^{4}+x^{3}+3\right)=0, \end{array}$$ (38)

gives *δ*_out_(*G*_1_) ≐ 0.683953. Following the same procedure, we get

$$\begin{array}{c} \alpha_{\text{in}}>2\;\text{and}\;\alpha_{\text{in}}<9. \end{array}$$ (39)

This leads to

$$\begin{array}{c} {P^{*}}_{G_{1},\text{in}}\left(x\right)=\alpha_{\text{in}}-P_{G_{1},\text{in}}\left(x\right)=3-\left(2x^{6}+2x^{5}+x^{4}+x^{3}+x^{2}+2\right)=0. \end{array}$$ (40)

Solving Eq (40) finally gives *δ*_in_ ≐ 0.608309. Similarly, for *G*_2_ in Fig (1),

$$P_{G_{2},\text{out}}\left(x\right)\;=\;8x+1,$$ (41)

$$P_{G_{2},\text{in}}\left(x\right)=8x+1.$$ (42)

We see that $P_{G_{2},\text{out}}(x)\;=\;P_{G_{2},\text{in}}(x)$. Also, we infer

$$\begin{array}{c} \alpha_{\text{out}}=\alpha_{\text{in}}>1\;\text{and}\;\alpha_{\text{out}}=\alpha_{\text{in}}<9. \end{array}$$ (43)

So, *α*_out_ = *α*_in_ ∈ {2, 3, 4, 5, 6, 7, 8}. Finally,

$$P^{*}{}_{G_{2},\text{out}}\left(x\right)\;=\;\alpha_{\text{out}}-P_{G_{2},\text{out}}\left(x\right)=2-\left(8x+1\right)=0,$$ (44)

$$P^{*}{}_{G_{2},\text{in}}\left(x\right)\;=\;\alpha_{\text{in}}-P_{G_{2},\text{in}}\left(x\right)=2-\left(8x+1\right)=0.$$ (45)

Hence, *δ*_out_ = *δ*_in_ ≐ 0.125. These findings are summarized in Table (1).

**Table 1:** Characteristics of the example graphs *G*_1_ and *G*_2_.

	Op.	*G*_1_ = (*V*, *E*), |*V*| = 9, |*E*| = 31		Op.	*G*_2_ = (*V*, *E*), |*V*| = 9, |*E*| = 8
*P*_G,out_(*x*)	=	2*x*^7^ + *x*^6^ + 2*x*^4^ + *x*^3^ + 3	*P*_G,out_(*x*)	=	8*x* + *x*
$P_{G,\text{out}}^{*}\left(x\right)$	=	1 − (2*x*^7^ + *x*^6^ + 2*x*^4^ + *x*^3^)	$P_{G,\text{out}}^{*}\left(x\right)$	=	1 − 8*x*
*α*_*out*_	∈	{4, 5, 6, 7, 8}	*α*_*out*_	∈	{2, 3, 4, 5, 6, 7, 8}
*P*_G,in_(*x*)	=	2*x*^6^ + 2*x*^5^ + *x*^4^ + *x*^3^ + *x*^2^ + 2	$P_{G,\text{in}}^{*}\left(x\right)$	=	8*x* + 1
$P_{G,\text{in}}^{*}\left(x\right)$	=	1 − (2*x*^6^ + 2*x*^5^ + *x*^4^ + *x*^3^ + *x*^2^)	$P_{G,\text{in}}^{*}\left(x\right)$	=	1 − 8*x*
*α*_*in*_	∈	{3, 4, 5, 6, 7, 8}	*α*_*in*_	∈	{2, 3, 4, 5, 6, 7, 8}
*I*_1_	=	0.683953	*I*_1_	=	0.125
*I*_2_	=	0.608309	*I*_2_	=	0.125
*I*_3_	=	0.646131	*I*_3_	=	0.125
*I*_4_	=	0.803478	*I*_4_	=	0.353553
*I*_5_	=	0.876937	*I*_5_	=	4.158883
*I*_7_	=	0.430555	*I*_7_	=	0.111111

Op.—operator

## 3 Numerical results

### 3.1 Software and computation

For the work of this paper, we used R [28] to generate the numerical results. To generate the classes of directed graphs, we used igraph and its functions [29], see Section (3.2). Also, we performed tests using igraph to ensure the graphs are pairwise non-isomorphic as well as connected. Moreover, the packages graph and QuACN were used to determine the in-degree and out-degree distribution of the generated graphs [30, 31].

### 3.2 Definition of graph classes

In this section, we define the graph classes we used for performing the numerical analysis. Note that we always performed 1000 repetitions when generating the graphs as we deal with random graphs.

**Definition 3.1** *The class* $G_{1}$ *contains* *20.000 directed*, *connected randomly chosen graphs* *G* = (*V*, *E*) *with 9 vertices*.

Note that we have relied on the Erdős—Rényi model [32] to generate these digraphs where min(|*E*|) = 8 and max(|*E*|) = 36. The number of edges and their direction were randomly selected.

**Definition 3.2** $G_{2}^{1}$ *contains 500 directed*, *connected*, *hierarchical graphs* *G* = (*V*, *E*) *which are randomly generated*. *The vertex and the edge sets are given by* [33]

$$\begin{array}{ll} V & =\;\{v_{01},\cdots \;,\;v_{0|V_{0}|},\;v_{11},\cdots \;,\;v_{1|V_{1}|},\;v_{21},\cdots \;,\;v_{2|V_{2}|},\;v_{31},\cdots \;,\;v_{3|V_{3}|}\},\\ E & \subseteq \;E_{1}\;\cup \;E_{2}. \end{array}$$ (46)

*These graphs have four levels*. *Level 0 is the root level*. |*V*_*i*_|, 0 ≤ *i* ≤ 3 *is the number of vertices on level* *i*. *E*_1_ *represents the set of edges which jump exactly one level*. *E*_2_ *is the set of jump edges which over-jump at least one level*. *Here*, *all edges move upwards*, *which means from level 3 to the root level*. *Thus*, 5 ≤ |*V*| ≤ 30.

**Fig 2:**
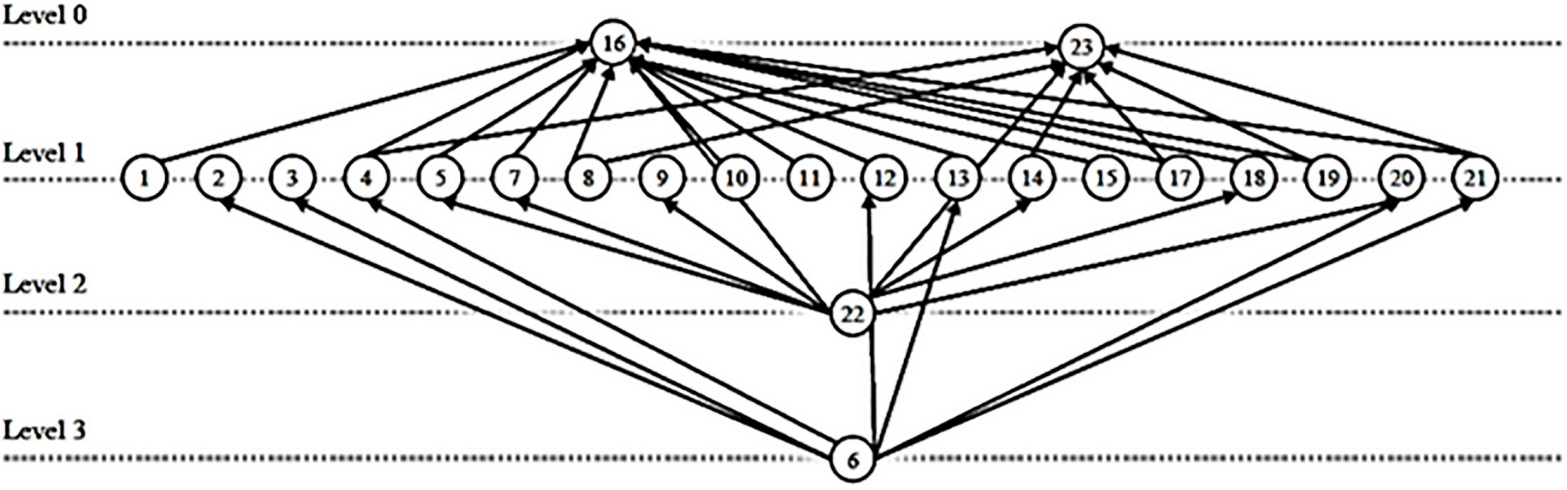
An example graph $G\in G_{2}^{1}$ where |*V*| = 23, |*E*| = 35.

Note that these graphs are usually called directed universal graphs and have been introduced in [33]. An example of such a graph is depicted in Fig (2).

**Definition 3.3** $G_{2}^{2}$ *contains 500 directed connected hierarchical graphs* *G* = (*V*, *E*) *based on Definition* *(3.2)*, *where* |*V*| = 20 *and* 8 ≤ |*E*| ≤ 30.

The reason we choose hierarchical (random) graphs for our analysis is that they appear in many real world applications, see [33]. Hierarchical graphs appear in many disciplines such as biology, management and manufacturing, see [34, 35]. Noteworthy are BOM-structures (Bill of Material) [34, 35]. These graphs have been widely used to analyze production systems and for representing optimizational tasks.

### 3.3 Correlation analysis

**Fig 3:**
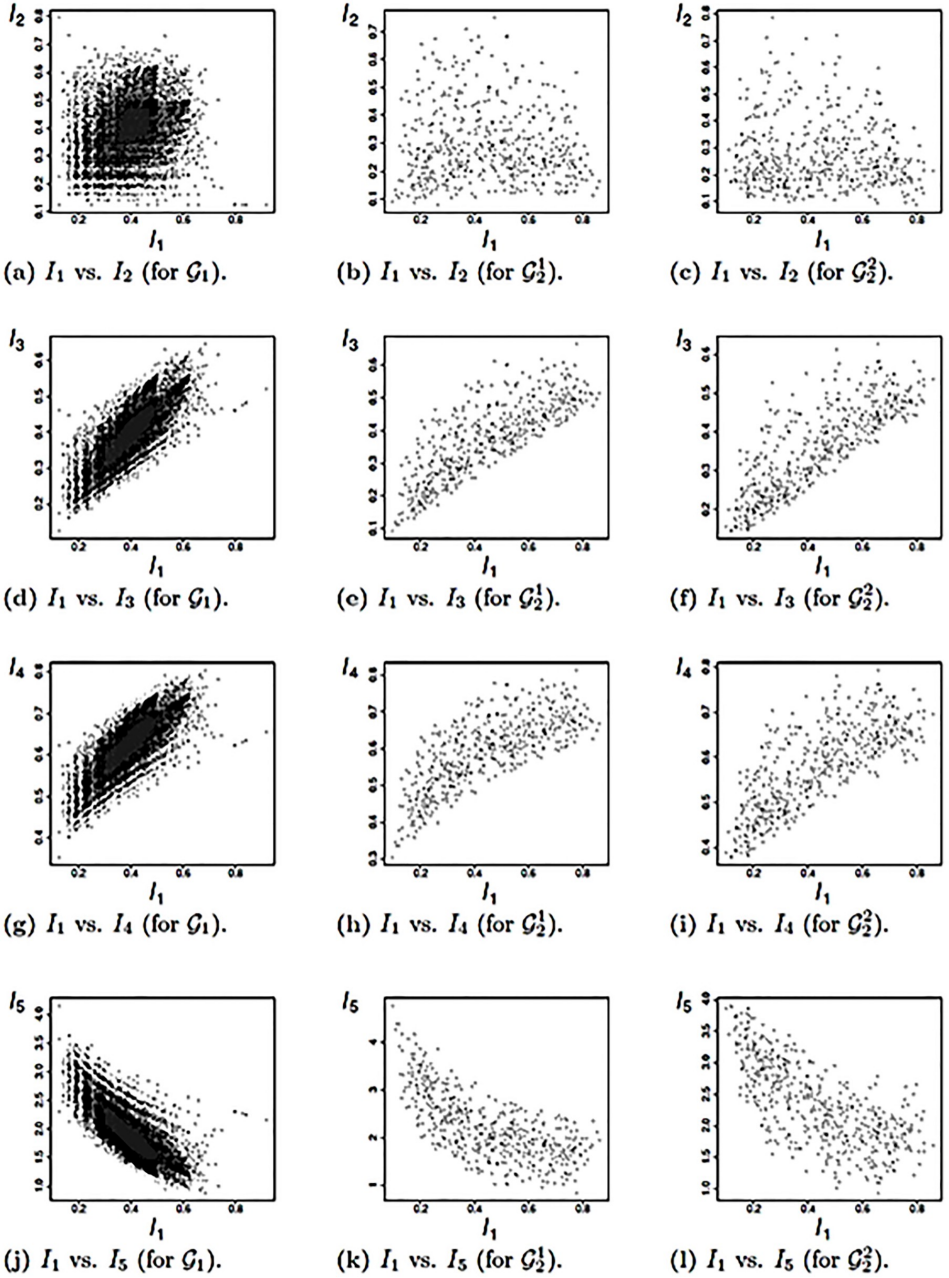
Scatter plots for *I*_1_ vs. *I*_*j*_, 2 ≤ *j* ≤ 5.

**Fig 4:**
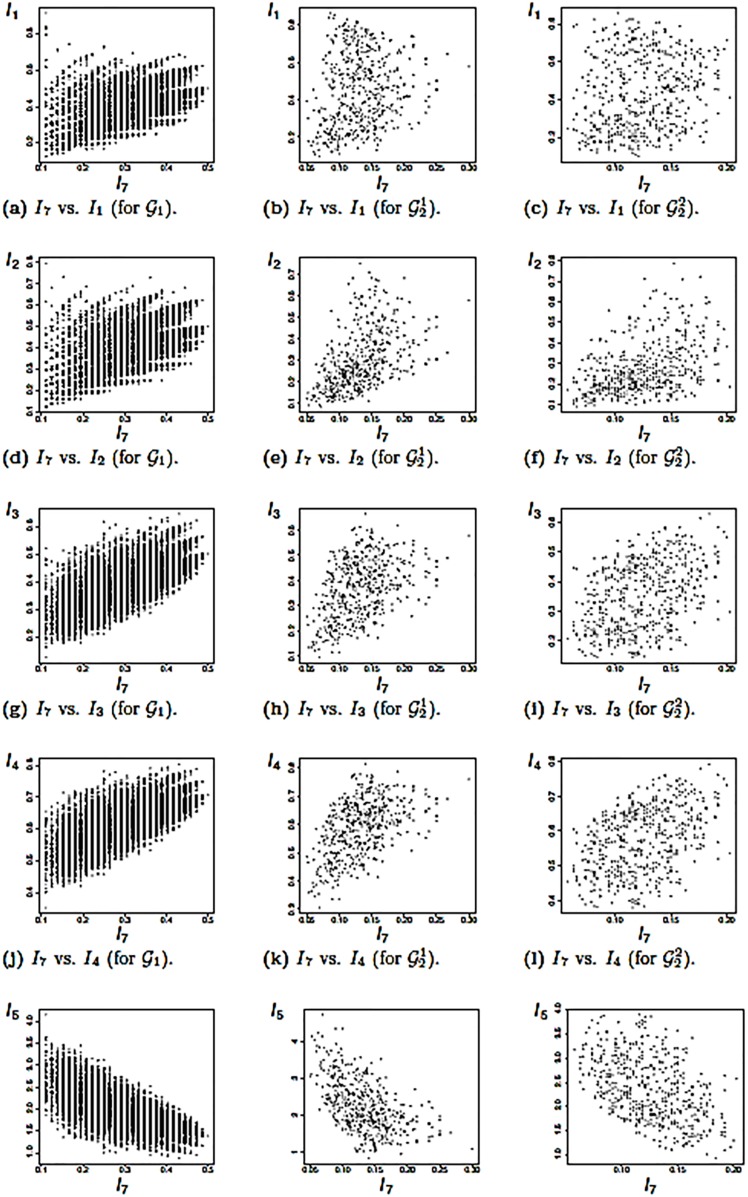
Scatter plots for *I*_7_ vs. *I*_*j*_, 2 ≤ *j* ≤ 5.

In this section, we discuss correlations between the graph measures applied to the classes of graphs defined above. The discussion is limited to the results shown in Figs (3) and (4). Other correlations have been found but are not presented explicitly here. We begin with results shown in Fig (3) for graph class $G_{1}$. Observe that there are many degenerate cases, i.e., non-isomorphic graphs having the same measure values. Interestingly, the two zeros of the polynomials P*_G,out_(*x*) and P*_G,in_(*x*) represented by the measures *I*_1_ and *I*_2_ are rather weakly correlated. See Fig (3a). Thus, these indices capture structural information differently on random graphs with 9 vertices. A plausible explanation for the values of the Spearman correlation being higher for the random graphs of $G_{1}$ (shown by Fig (3d), (3g) and (3h) is that *I*_3_, *I*_4_, *I*_5_ also depend on *I*_1_. For some combinations of *I*_1_ vs. *I*_*j*_, 2 ≤ *j* ≤ 5, the correlation is also weak and, hence, the associated measures give quite different values. For instance, this is the case for *I*_1_ and *I*_2_ on $G_{2}^{1}$. A possible reason for the weak correlation and for the wide spread shown on the scatter plots is that the underlying hierarchical graphs have a more distinct structure compared to the completely random graphs $\in G_{1}$. This also implies that the degeneracy is much lower compared with $\in G_{1}$, which also holds for the scatter plots shown in Fig (3) for $G_{2}^{2}$. All the values of the Spearman correlation are given in Table (2).

**Table 2:** Spearman correlation of the graph measures for $G_{1}$, $G_{2}^{1}$ and $G_{2}^{2}$.

$G_{1}$						$G_{2}^{1}$						$G_{2}^{2}$					
	*I*_1_	*I*_2_	*I*_3_	*I*_4_	*I*_5_		*I*_1_	*I*_2_	*I*_3_	*I*_4_	*I*_5_		*I*_1_	*I*_2_	*I*_3_	*I*_4_	*I*_5_
*I*_2_	0.3709					*I*_2_	0.0046					*I*_2_	-0.0146				
*I*_3_	0.8221	0.8194				*I*_3_	0.8259	0.5106				*I*_3_	0.8403	0.4818			
*I*_4_	0.8203	0.8199	0.9989			*I*_4_	0.7735	0.5956	0.9898			*I*_4_	0.7948	0.5626	0.9911		
*I*_5_	-0.8171	-0.8188	-0.9959	-0.9990		*I*_5_	-0.7013	-0.6794	-0.9559	-0.9873		*I*_5_	-0.7316	-0.6422	-0.9626	-0.9894	
*I*_7_	0.6112	0.6218	0.7439	0.7541	-0.7624	*I*_7_	0.0140	-0.0666	-0.0135	-0.0215	0.0286	*I*_7_	0.1825	0.4427	0.3741	0.4168	-0.4570

Similar results are obtained for all other combinations of *I*_*i*_ vs. *I*_*j*_, 2 ≤ *j* ≤ 5, 2 ≤ *i* ≤ 6, which is the reason they are not shown explicitly here. Finally, consider Fig (4). These scatter plots as well as the values in Table (2), show that the edge density *I*_7_ has no structural relationship with all other measures. The left column of Fig (4), also shows that *I*_7_ is highly degenerated. This is not surprising given its definition. As indicated earlier, the measures applied to graph classes containing hierarchical graphs have fewer degeneracies.

### 3.4 Extremal graphs/relations

**Fig 5:**
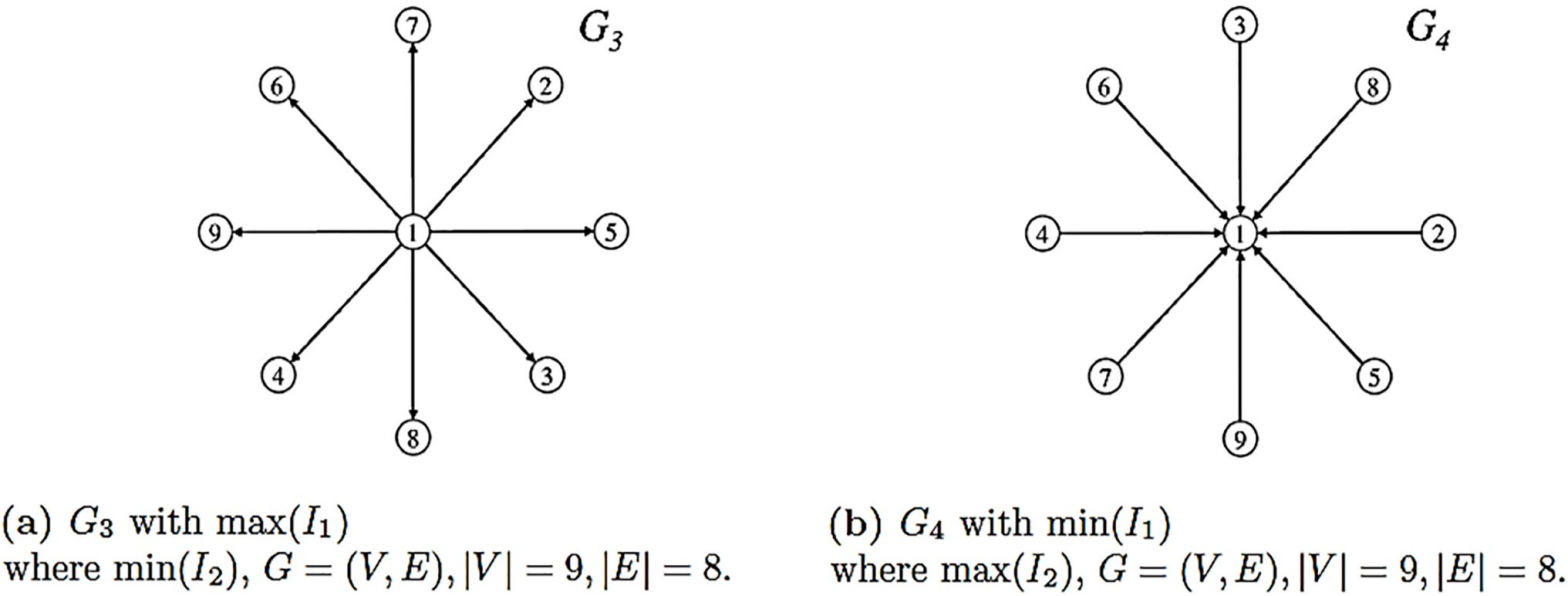
Two graphs $\in G_{1}$ maximizing and minimizing *I*_1_ and *I*_2_.

Now we take a closer look at graphs that attain maximum or minimum values under the graph measures of Definition (2.6). Fig (5) shows two graphs for which max(*I*_1_) and min(*I*_1_) obtains. Take graph *G*_3_ as an example. To calculate $P_{G_{3},\text{out}}^{*}\left(x\right)$, we apply Theorem (2.1) and solve the equation

$$\begin{array}{c} \alpha_{\text{out}}-\left(x^{8}+8\right)=0. \end{array}$$ (47)

According to Theorem (2.1), we know that Eq (47) has a unique, positive zero in the interval (0, 1) which obviously depends on the parameter *α*_out_. Solving Eq (47) gives $x=\sqrt[{8}]{\alpha_{\text{out}}-8}$. So, to maximize graph measure *I*_1_ we have to determine $\max_{\alpha_{\text{out}}}\left\{\sqrt[{8}]{\alpha_{\text{out}}-8}\right\}.$ Note that Theorem (2.1) also gives 8 < *α*_out_ < 9. In Table (3), we set *α*_out_ = 8.5 in order to compute the values of the graph complexity measures. Clearly, this is the maximum value of *I*_1_ for the given parameter. The case min(*I*_1_) for *G*_4_ can be shown analogously.

**Table 3:** Polynomials, parameters and graph measures for calculating *I*_1_ and *I*_2_ for *G*_3_, $G_{4}\in G_{1}$.

	Op.	*G*_3_ = (*V*, *E*), |*V*| = 9, |*E*| = 8		Op.	*G*_4_ = (*V*, *E*), |*V*| = 9, |*E*| = 8
*P*_G,out_(*x*)	=	1*x*^8^ + 8	*P*_G,out_(*x*)	=	8*x* + 1
$P_{G,\text{out}}^{*}\left(x\right)$	=	0.5 − *x*^8^	$P_{G,\text{out}}^{*}\left(x\right)$	=	1 − 8*x*
*α*_out_	∈	{8.5}	*α*_out_	∈	{2, 3, 4, 5, 6, 7, 8}
*P*_G,in_(*x*)	=	8*x* + 1	*P*_G,in_(*x*)	=	*x*^7^ + *x* + 7
$P_{G,\text{in}}^{*}\left(x\right)$	=	1 − 8*x*	$P_{G,\text{in}}^{*}\left(x\right)$	=	1 − (*x*^7^ + *x*)
*α*_*in*_	∈	{2, 3, 4, 5, 6, 7, 8}	*α*_*in*_	∈	{8}
*I*_1_	=	0.917004	*I*_1_	=	0.125
*I*_2_	=	0.125	*I*_2_	=	0.796544
*I*_3_	=	0.521002	*I*_3_	=	0.460772
*I*_4_	=	0.655578	*I*_4_	=	0.623023
*I*_5_	=	2.166084	*I*_5_	=	2.306914
*I*_7_	=	0.111111	*I*_7_	=	0.111111

Op.—operator

Now, we are in position to begin our analysis of extremal conditions and relations between digraphs.

**Theorem 3.1** *Let* *G* = (*V*_*G*_, *E*_*G*_) *and* *H* = (*V*_*H*_, *E*_*H*_), *be two digraphs*. *Define*

$$P_{G}\left(x\right)\;≔\alpha -\left(a_{1}^{G}x+a_{0}^{G}\right),\;a_{i}^{G}\in \mathbb{N},$$ (48)

$$P_{H}\left(x\right)\;≔\alpha -\left(a_{n}^{H}x^{n}+a_{n-1}^{H}x^{n-1}+\cdots +a_{1}^{H}x+a_{0}^{H}\right),\;a_{i}^{H}\in \mathbb{N},$$ (49)

*and assume that there exist graphs* *G* *and* *H* *with the given polynomials*. *Also*, *assume that the conditions of Theorem* *(2.1)*, *namely*,

$$a_{0}^{G}\;<\alpha <a_{1}^{G}+a_{0}^{G},$$ (50)

$$a_{0}^{H}\;<\alpha <a_{n}^{H}+a_{n-1}^{H}+\cdots +a_{1}^{H}+a_{0}^{H},$$ (51)

*are satisfied*, *and there exists* *α* *satisfying the Inequalities* (50) *and* (51). *The equation*

$$\begin{array}{c} \frac{\alpha -a_{0}^{G}}{a_{1}^{G}}=\delta^{\alpha }\left(G\right)>\delta^{\alpha }\left(H\right), \end{array}$$ (52)

*holds if*

$$\begin{array}{c} \alpha <\frac{a_{n}^{H}}{{\left(a_{1}^{G}\right)}^{n}}{\left(\alpha -a_{0}^{G}\right)}^{n}+\frac{a_{n-1}^{H}}{{\left(a_{1}^{G}\right)}^{n-1}}{\left(\alpha -a_{0}^{G}\right)}^{n-1}+\cdots +\frac{a_{1}^{H}}{a_{1}^{G}}\left(\alpha -a_{0}^{G}\right)+a_{0}^{H}. \end{array}$$ (53)

**Proof**: Consider the polynomials represented by the Eqs (48) and (49). Since we assume the Inequalities (50) and (50) are valid, Theorem (2.1) assures us that *δ*^*α*^(*G*) and *δ*^*α*^(*H*) lie in the interval (0, 1). The notation *δ*^*α*^ makes explicit the dependence of the zeros on the parameter *α*. Clearly, $P_{G}\left(x\right)=\alpha -\left(a_{1}^{G}x+a_{0}^{G}\right)=0$ implies $\delta^{\alpha }\left(G\right)=\frac{\alpha -a_{0}^{G}}{a_{1}^{G}}$. Assuming *P*_*H*_(*δ*^*α*^(*G*)) < 0, we conclude that Inequality (52) must be satisfied. But

$$\begin{array}{c} P_{H}\left(\delta^{\alpha }\left(G\right)\right)=\alpha -\left[\frac{a_{n}^{H}}{{\left(a_{1}^{G}\right)}^{n}}{\left(\alpha -a_{0}^{G}\right)}^{n}+\frac{a_{n-1}^{H}}{{\left(a_{1}^{G}\right)}^{n-1}}{\left(\alpha -a_{0}^{G}\right)}^{n-1}+\cdots +\frac{a_{1}^{H}}{a_{1}^{G}}\left(\alpha -a_{0}^{G}\right)+a_{0}^{H}\right]<0, \end{array}$$ (54)

implies the Inequality (53).

**Corollary 3.1** *Referring to Theorem* *(3.1)*, *we assume that for the two polynomials represented by the* Eqs (48) and (49) *the Inequalities* (50) and (50) *are satisfied*. *If* $a_{1}^{H}=a_{1}^{G}$ and $a_{0}^{G}=a_{0}^{H}$, *then Inequality* (52) *always holds*.

**Proof**: Setting $a_{1}^{H}=a_{1}^{G}$ and $a_{0}^{G}=a_{0}^{H}$ in Inequality (53), we obtain

$$\begin{array}{c} \frac{a_{n}^{H}}{{\left(a_{1}^{G}\right)}^{n}}{\left(\alpha -a_{0}^{G}\right)}^{n}+\frac{a_{n-1}^{H}}{{\left(a_{1}^{G}\right)}^{n-1}}{\left(\alpha -a_{0}^{G}\right)}^{n-1}+\cdots +\frac{a_{2}^{H}}{{\left(a_{1}^{G}\right)}^{2}}{\left(\alpha -a_{0}^{G}\right)}^{2}>0. \end{array}$$ (55)

As $\alpha >a_{0}^{G}$, Inequality (55) is always satisfied.

Finally, we state the following theorem.

**Theorem 3.2** *Let* *G* = (*V*_*G*_, *E*_*G*_) *and* *H* = (*V*_*H*_, *E*_*H*_) *be two digraphs*, *and define*

$$P_{G}\left(x\right)\;≔\alpha -\left(a_{n}^{G}x^{n}+a_{n-1}^{G}x^{n-1}+\cdots +a_{1}^{G}x+a_{0}^{G}\right),\;a_{i}^{G}\in \mathbb{N}),$$ (56)

$$P_{H}\left(x\right)\;≔\alpha -\left(a_{n}^{H}x^{n}+a_{n-1}^{H}x^{n-1}+\cdots +a_{1}^{H}x+a_{0}^{H}\right),\;a_{i}^{H}\in \mathbb{N}.$$ (57)

*assuming that there are graphs* *G* *and* *H* *with the given polynomials*. *Now*,

$$a_{0}^{G}\;<\alpha <a_{n}^{G}+\cdots +a_{1}^{G}+a_{0}^{G},$$ (58)

$$a_{0}^{H}\;<\alpha <a_{n}^{H}+\cdots +a_{1}^{H}+a_{0}^{H}.$$ (59)

*Choose* *α* *such that it satisfies the Inequalities* (58) and (59). *If*

$$\begin{array}{c} a_{n}^{G}<a_{i}^{H}\;for\;1\leq i\leq n, \end{array}$$ (60)

*then*

$$\begin{array}{c} \delta^{\alpha }\left(G\right)>\delta^{\alpha }\left(H\right). \end{array}$$ (61)

*Both* *δ*^*α*^(*G*) *and* *δ*^*α*^(*H*) *lie in* (0, 1).

**Proof**: From the Inequality-System (60), we derive

$$\begin{array}{c} P_{G}\left(x\right)=\alpha -\left(a_{n}^{G}x^{n}+a_{n-1}^{G}x^{n-1}+\cdots +a_{1}^{G}x+a_{0}^{G}\right)\\ \;>\alpha -\left(a_{n}^{H}x^{n}+a_{n-1}^{H}x^{n-1}+\cdots +a_{1}^{H}x+a_{0}^{H}\right)=P_{H}\left(x\right). \end{array}$$ (62)

But *P*_*G*_(*x*) > *P*_*H*_(*x*) implies Inequality (61).

Consider the two graphs *G*_4_ (see Fig (5)) and *G* (see Fig (2)). We use these examples to demonstrate Theorem (3.1). In this demonstration, we consider only the out-degree polynomial and obtain

$$P^{*}{}_{G_{4},\text{out}}\left(x\right)\;=\alpha -\left(8x+1\right),$$ (63)

$$P^{*}{}_{G,\text{out}}\left(x\right)\;=\alpha -\left(x^{8}+x^{7}+5x^{2}+10x+6\right).$$ (64)

Note that *G*_4_ and *G*_2_ (see Fig (1) and Table (1)) have the same polynomial $P_{G_{4},\text{out}}(x)$ and $P_{G_{2},\text{out}}(x)$ but the two graphs are non-isomorphic. We call such polynomials *degenerate*. The Inequalities (26) and (28) give for *G*_4_, 1 < *α* < 9 and for *G*, 6 < *α* < 23. So, if we choose, e.g., *α* = 7, these two inequalities are satisfied. So, we need to check whether the Inequality (53)

$$\begin{array}{c} 7<\frac{1}{8^{8}}{\left(7-1\right)}^{8}+\frac{1}{8^{7}}{\left(7-1\right)}^{7}+\frac{5}{8^{5}}{\left(7-1\right)}^{5}+\frac{10}{8}\left(7-1\right)+6, \end{array}$$ (65)

holds in this case. Since 7 < 14.920, Theorem (3.1) says

$$\begin{array}{c} \delta^{\alpha =7}\left(G_{4}\right)>\delta^{\alpha =7}\left(G\right). \end{array}$$ (66)

From Eqs (63) and (64) with *α* = 7, we finally obtain

$$\begin{array}{c} \delta^{\alpha =7}\left(G_{4}\right)=\frac{3}{4}\;\text{and}\;\delta^{\alpha =7}\left(G\right)≐0.099995. \end{array}$$ (67)

**Fig 6:**
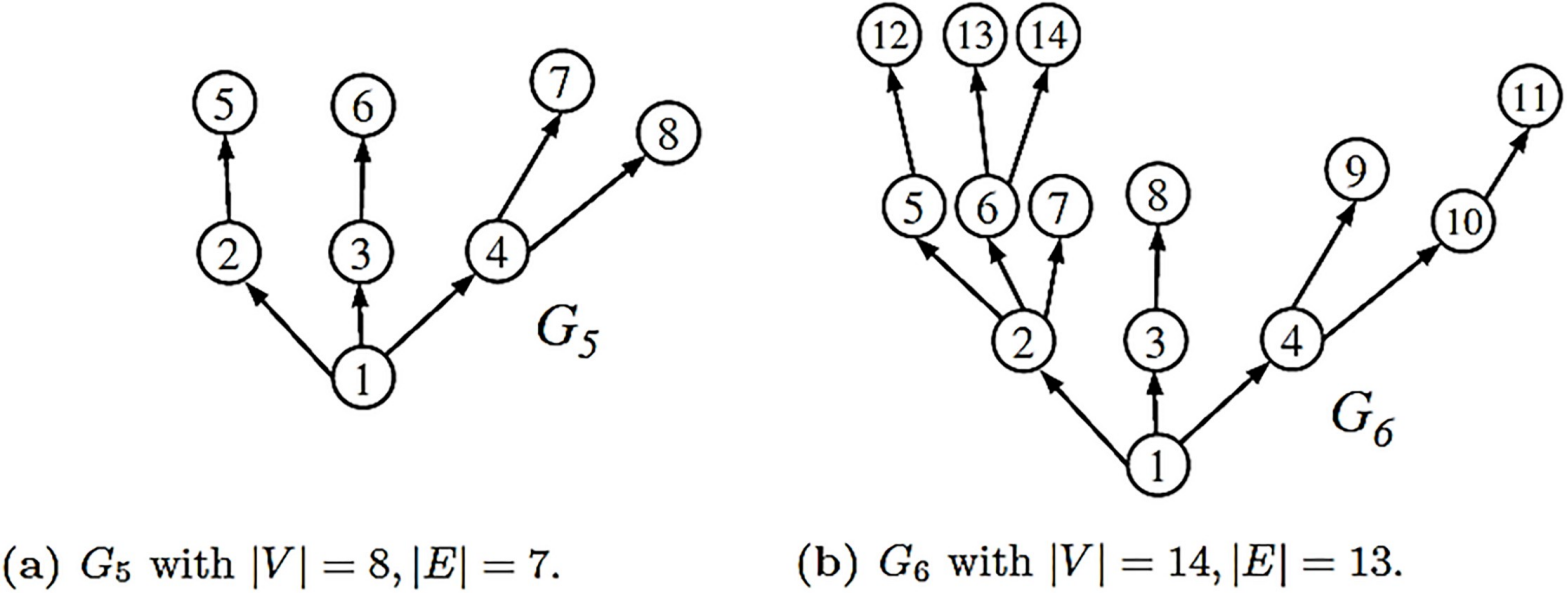
The two example graphs *G*_5_ and *G*_6_.

The two graphs shown by Fig (6A) provide another illustration of Theorem (3.2). Again, we deal only with the out-degree polynomials. From Fig (6), we determine

$$P^{*}{}_{G_{5},\text{out}}\left(x\right)\;=\alpha -\left(x^{3}+x^{2}+2x+4\right),$$ (68)

$$P^{*}{}_{G_{6},\text{out}}\left(x\right)\;=\;\alpha -\left(2x^{3}+2x^{2}+3x+6\right).$$ (69)

The two graphs have a different number of vertices and edges but their underlying out-degree polynomials have the same degree. Moreover, the Inequality-System (60) is satisfied. For Inequalities 4 < *α* < 8 for *G*_5_ and 6 < *α* < 13, we can choose *α* = 7. Calculating the zeros of the polynomials represented by the Eqs (68) and (69) gives

$$\begin{array}{c} \delta^{\alpha =7}\left(G_{5}\right)≐0.843734>\delta^{\alpha =7}\left(G_{6}\right)≐0.271069. \end{array}$$ (70)

### 3.5 Homogeneity of The Zeros—Influence of *α*_out_ and *α*_in_

In this section, we briefly investigate the influence of *α*_*out*_ and *α*_*in*_. To measure the divergences between the resulting zeros, we define a homogeneity measure.

For this discussion we restrict attention to out-degrees; analogous results hold for in-degrees. Let *G* be a directed graph with associated polynomials *P*_*G*,out_(*x*) and P*_G,out_(*x*). Suppose the Inequalities (26) and (28), yield $k_{\text{out}}^{G}$ possible values $\alpha_{\text{out}}^{G,1},\cdots ,\alpha_{\text{out}}^{G,k_{\text{out}}^{G}}$. Thus, $\delta_{\text{out}}^{G,1},\delta_{\text{out}}^{G,2},\cdots ,\delta_{\text{out}}^{G,k_{\text{out}}^{G}}$ are possible roots of P*_G,out_(*x*) = 0.

To define the homogeneity of the set

$$\begin{array}{c} S_{\text{out}}^{G}=\left\{\delta_{\text{out}}^{G,1},\delta_{\text{out}}^{G,2},\cdots ,\delta_{\text{out}}^{G,k_{\text{out}}^{G}}\right\}. \end{array}$$ (71)

we use a real valued distance measure, namely,

$$\begin{array}{c} d(x,y)=|x-y|,x,y\in R. \end{array}$$ (72)

The homogeneity of $S_{\text{out}}^{G}$ is defined by

$$\begin{array}{c} h\left(S_{\text{out}}^{G}\right)=\frac{1}{k_{\text{out}}^{G}\left(k_{\text{out}}^{G}-1\right)}\sum_{i=1}^{k_{\text{out}}^{G}}\sum_{j=1}^{k_{\text{out}}^{G}}d\left(\delta_{\text{out}}^{G,i},\delta_{\text{out}}^{G,j}\right). \end{array}$$ (73)

**Fig 7:**
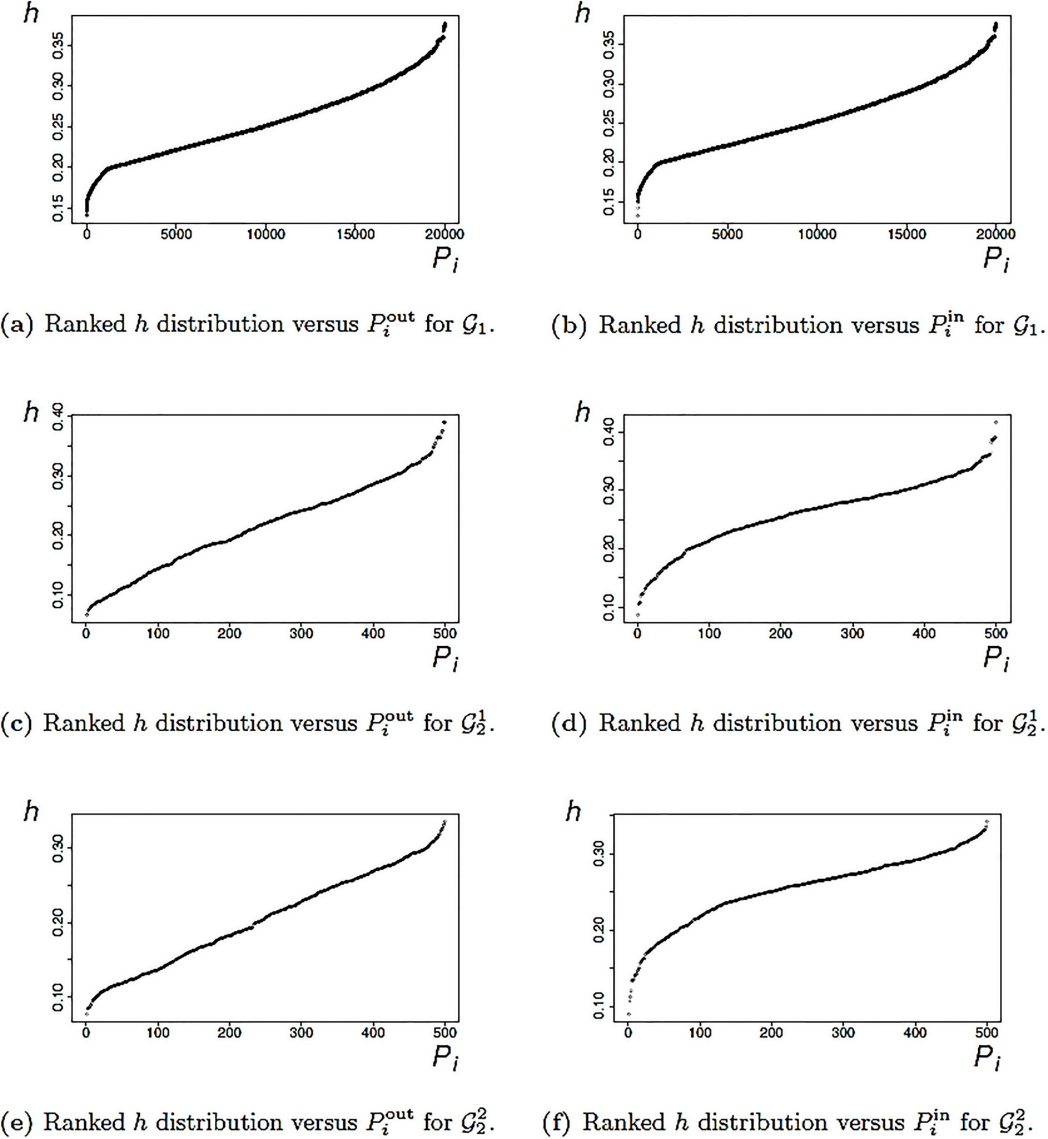
Distributions of the $h(S_{\text{out}}^{G})$ and $h(S_{\text{in}}^{G})$ values for all three graph classes.

The value for in-degrees is defined similarly. A high *h*-score indicates that the set *S*^*G*^ is inhomogeneous while a small value of *h* gives a high homogeneity rank of *S*^*G*^. The definition is illustrated in Fig (7).

The homogeneity values are plotted against the number of polynomials in Fig (7). First, observe that the distributions of the homogeneity values for out-degrees and in-degrees look very similar. Also, we observe that the differences between the zeros is quite small, which implies that homogeneity is high. This can be seen from the value-range in Fig (7). This result is not surprising as we explained in Section (2.1), i.e., the zeros of a polynomial are continuous functions of the coefficients of the polynomial. In fact, if we vary *α*_out_ or *α*_in_, we see that the coefficients of the resulting polynomials are quite similar, when only the constant terms *α*_out_ and *α*_in_ are changed, see the Eqs (22) and (23). Therefore, the small differences between the roots (and the high homogeneity values) reflect the continuity theorem for complex and real polynomials, see [22].

### 3.6 Computational complexity

In this section, we briefly sketch some ideas to determine the computational complexity to compute *δ*. Note that calculating the vertex degrees requires polynomial time, i.e., *O*(*n*^2^) in case *n* is the order of an input graph. Assigning the out- and in-degrees to the monomials *x*^*i*^ can be achieved in constant time and adding up those terms requires linear time complexity, i.e., *O*(*k*); *k* is the degree of the polynomial. Altogether, we see that we are able to construct an efficient algorithm to compute *δ*.

## 4 Summary and conclusion

In this paper, we have introduced new complexity measures for real networks. One reason for developing an alternative to degree-based measures such as the Zagreb indices [4] or entropies based on vertex degrees [36] is their high degeneracy, meaning that many pairwise non-isomorphic graphs have the same measured value. Similar to [37], we developed a polynomial-based approach for measuring the complexity of directed graphs. To the best of our knowledge, there are very few measures for directed graphs, e.g., treewith and girth, and these are true complexity measures for encoding structural information of a directed graph. Following [37], we developed graph polynomials based on the out- and in-degrees of directed graphs and constructed modified polynomials which posses a unique, positive zero in the interval (0, 1), depending on up to two parameters *α*_out_ and *α*_out_. These zeros *δ*_out_ and *δ*_in_ can be interpreted as complexity measures. Interestingly, we start with a graph invariant and construct polynomials which are associated with the graph. However, the graph measures defined here are algebraic quantities representing the zeros of polynomials.

Analytical results showing relationships between the graph measures have been demonstrated; we have obtained numerical results that show correlations between the graph measures, and have investigated the homogeneity of the zeros (graph measures). We compared our graph measures with the well-known edge density and found that our measures capture structural information differently. Also some of our measures seem to have useful properties, e.g., they possess a high discrimination power for graphs with a distinct graph topology. Our approach to analyzing the complexity of directed graphs is promising in that low computational complexity (i.e., vertex degrees of a directed graph can be determined in polynomial time) allows for applying the polynomial based measures to large networks.

As part of our ongoing research, we plan to continue investigating extremal properties of the measures. Also, we should like to perform a correlation analysis with other measures on a large scale, if we can find ones that can be computed in polynomial time. Existing measures based on game theory are computationally complex, see, e.g., [13].
